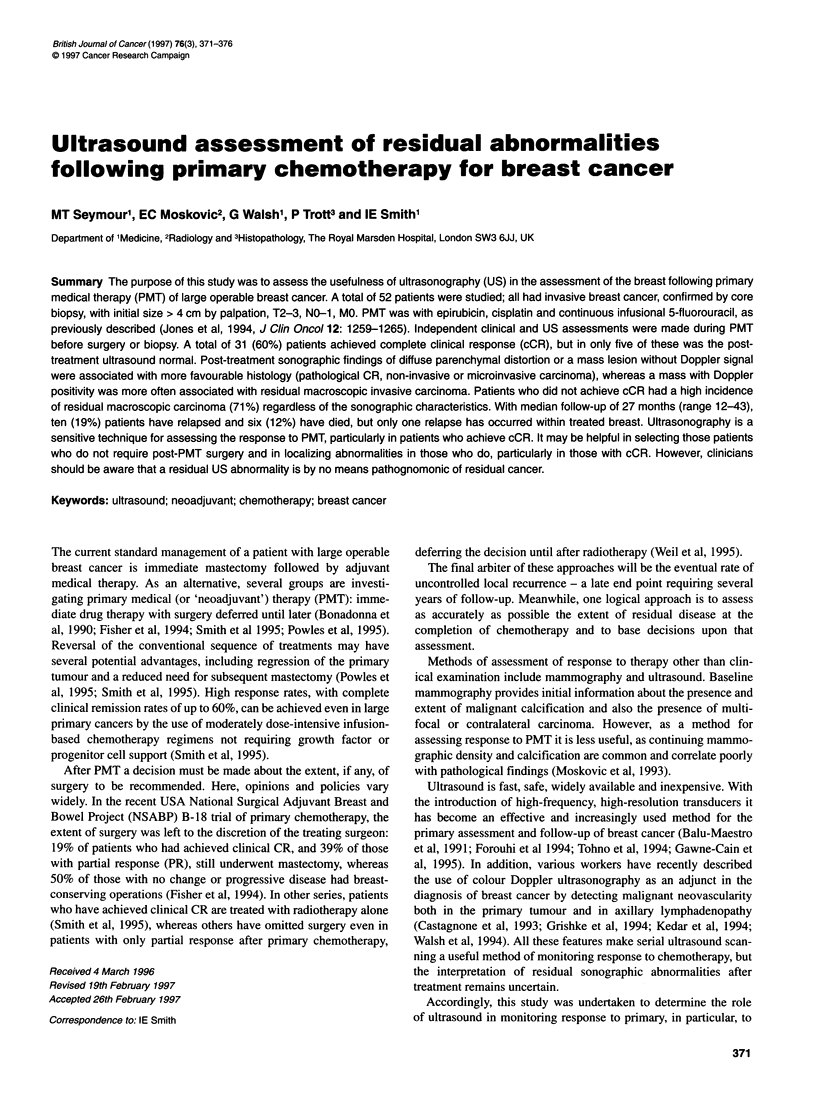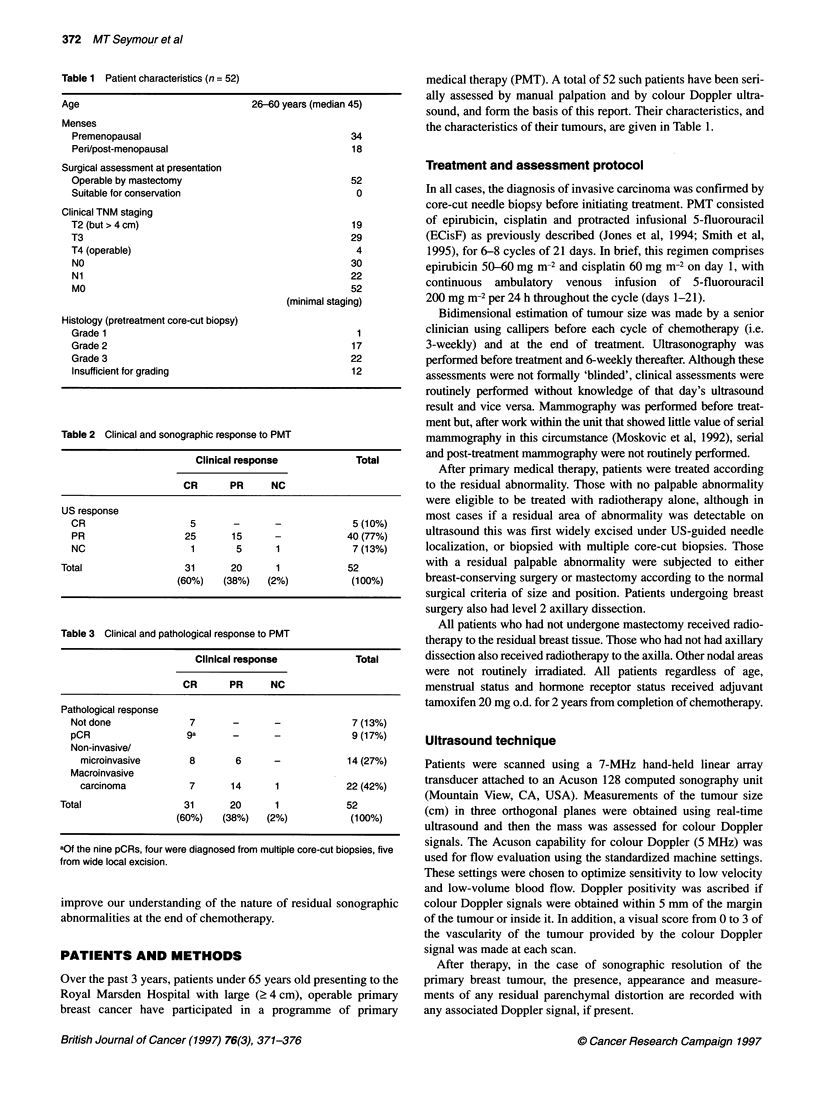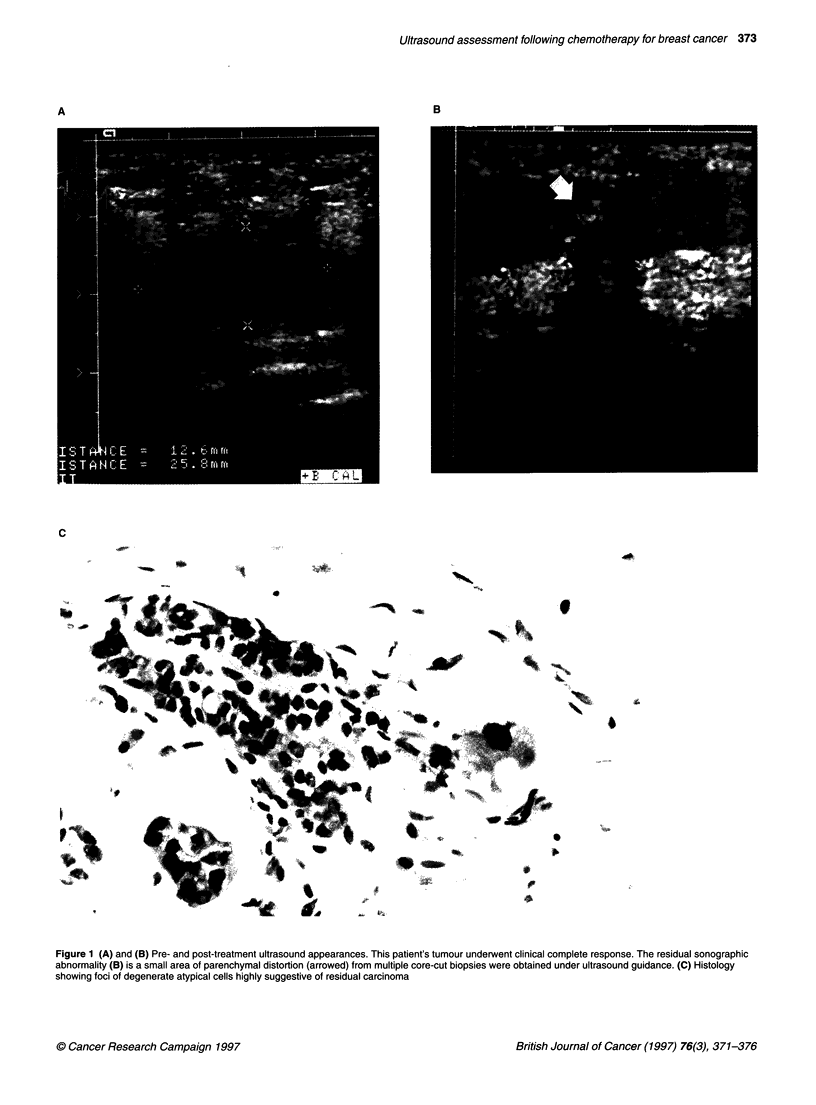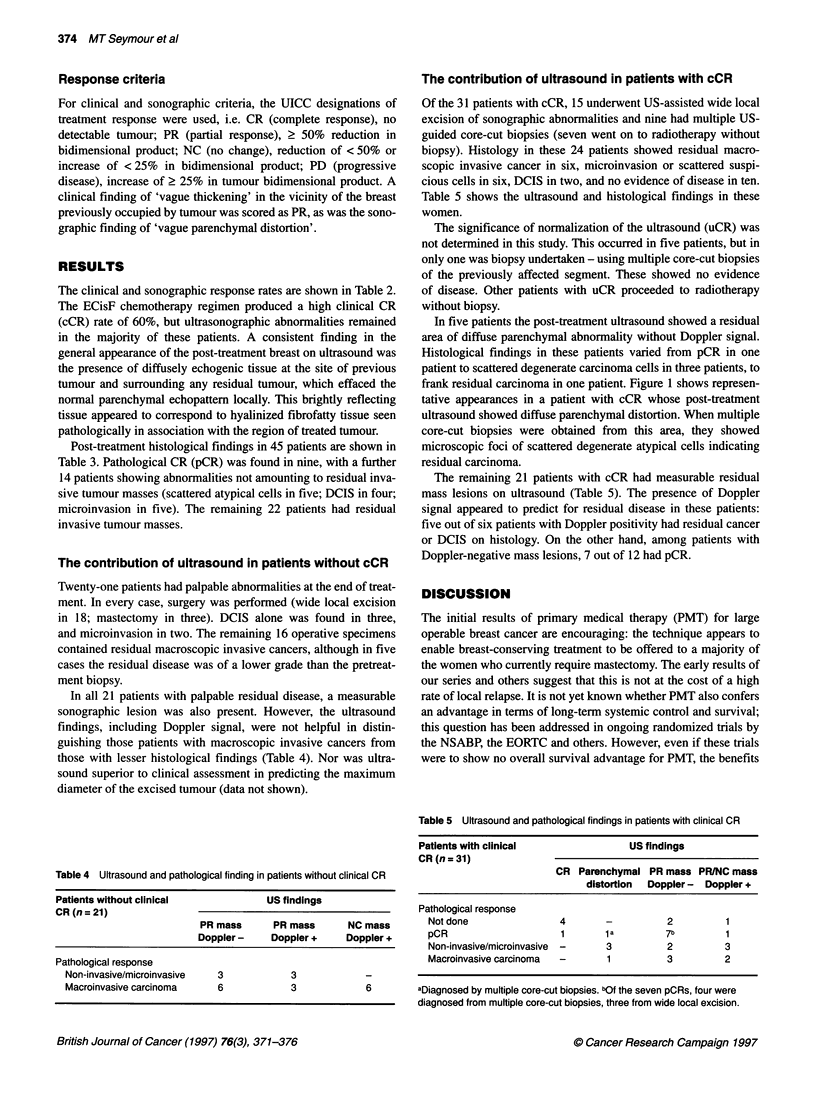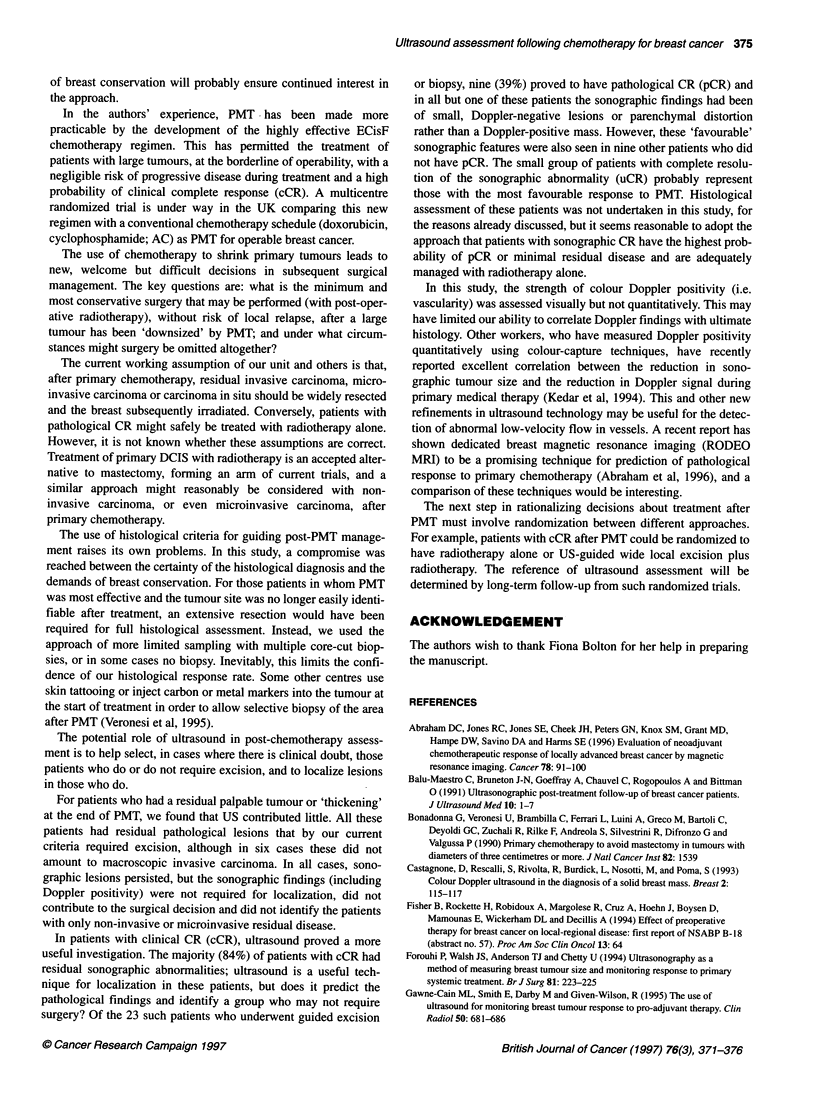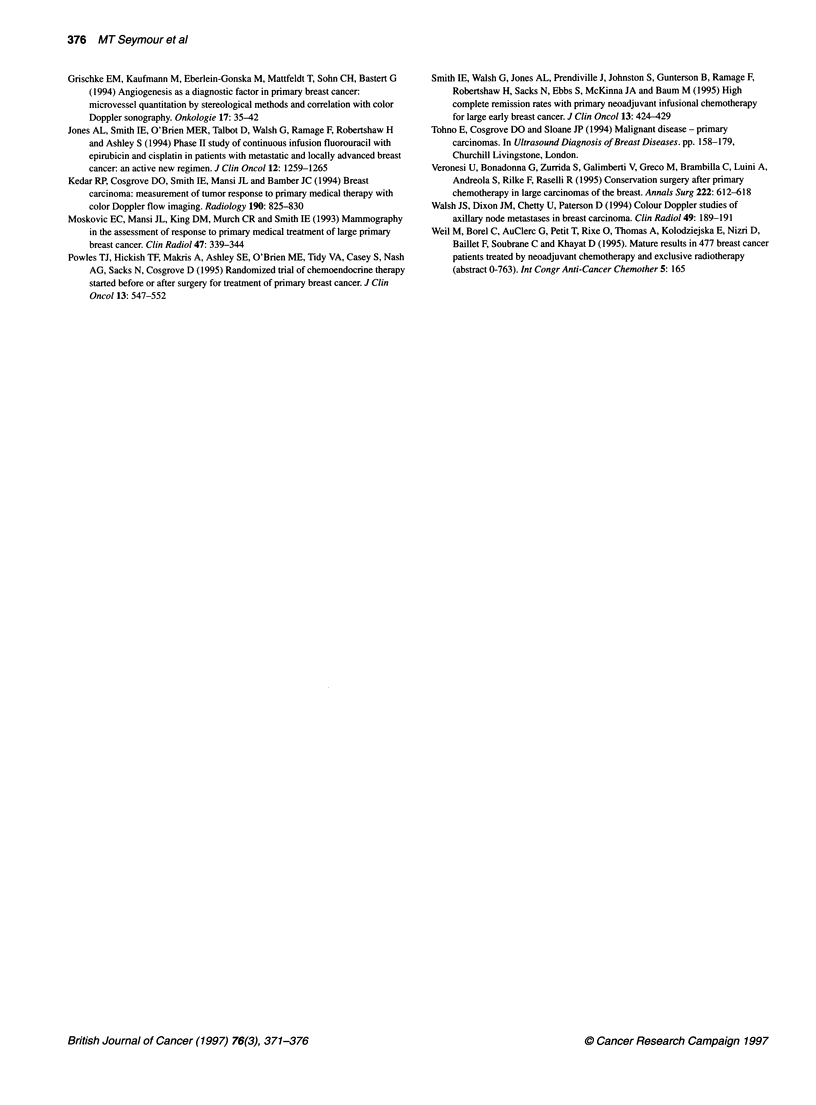# Ultrasound assessment of residual abnormalities following primary chemotherapy for breast cancer.

**DOI:** 10.1038/bjc.1997.392

**Published:** 1997

**Authors:** M. T. Seymour, E. C. Moskovic, G. Walsh, P. Trott, I. E. Smith

**Affiliations:** Department of Medicine, The Royal Marsden Hospital, London, UK.

## Abstract

**Images:**


British Journal of Cancer (1997) 76(3), 371–376
© 1997 Cancer Research Campaign

### Ultrasound assessment of residual abnormalities following primary chemotherapy for breast cancer

**MT Seymour[1], EC Moskovic[2], G Walsh[1], P Trott[3] and IE Smith[1]**

Department of [1]Medicine, [2]Radiology and [3]Histopathology, The Royal Marsden Hospital, London SW3 6JJ, UK

**Summary** The purpose of this study was to assess the usefulness of ultrasonography (US) in the assessment of the breast following primary medical therapy (PMT) of large operable breast cancer. A total of 52 patients were studied; all had invasive breast cancer, confirmed by core biopsy, with initial size > 4 cm by palpation, T2–3, N0–1, M0. PMT was with epirubicin, cisplatin and continuous infusional 5-fluorouracil, as previously described (Jones et al, 1994, *J Clin Oncol* **12**: 1259–1265). Independent clinical and US assessments were made during PMT before surgery or biopsy. A total of 31 (60%) patients achieved complete clinical response (cCR), but in only five of these was the post-treatment ultrasound normal. Post-treatment sonographic findings of diffuse parenchymal distortion or a mass lesion without Doppler signal were associated with more favourable histology (pathological CR, non-invasive or microinvasive carcinoma), whereas a mass with Doppler positivity was more often associated with residual macroscopic invasive carcinoma. Patients who did not achieve cCR had a high incidence of residual macroscopic carcinoma (71%) regardless of the sonographic characteristics. With median follow-up of 27 months (range 12–43), ten (19%) patients have relapsed and six (12%) have died, but only one relapse has occurred within treated breast. Ultrasonography is a sensitive technique for assessing the response to PMT, particularly in patients who achieve cCR. It may be helpful in selecting those patients who do not require post-PMT surgery and in localizing abnormalities in those who do, particularly in those with cCR. However, clinicians should be aware that a residual US abnormality is by no means pathognomonic of residual cancer.

**Keywords:** ultrasound; neoadjuvant; chemotherapy; breast cancer

The current standard management of a patient with large operable breast cancer is immediate mastectomy followed by adjuvant medical therapy. As an alternative, several groups are investigating primary medical (or 'neoadjuvant') therapy (PMT): immediate drug therapy with surgery deferred until later (Bonadonna et al, 1990; Fisher et al, 1994; Smith et al 1995; Powles et al, 1995). Reversal of the conventional sequence of treatments may have several potential advantages, including regression of the primary tumour and a reduced need for subsequent mastectomy (Powles et al, 1995; Smith et al, 1995). High response rates, with complete clinical remission rates of up to 60%, can be achieved even in large primary cancers by the use of moderately dose-intensive infusion-based chemotherapy regimens not requiring growth factor or progenitor cell support (Smith et al, 1995).

After PMT a decision must be made about the extent, if any, of surgery to be recommended. Here, opinions and policies vary widely. In the recent USA National Surgical Adjuvant Breast and Bowel Project (NSABP) B-18 trial of primary chemotherapy, the extent of surgery was left to the discretion of the treating surgeon: 19% of patients who had achieved clinical CR, and 39% of those with partial response (PR), still underwent mastectomy, whereas 50% of those with no change or progressive disease had breast-conserving operations (Fisher et al, 1994). In other series, patients who have achieved clinical CR are treated with radiotherapy alone (Smith et al, 1995), whereas others have omitted surgery even in patients with only partial response after primary chemotherapy, deferring the decision until after radiotherapy (Weil et al, 1995).

The final arbiter of these approaches will be the eventual rate of uncontrolled local recurrence – a late end point requiring several years of follow-up. Meanwhile, one logical approach is to assess as accurately as possible the extent of residual disease at the completion of chemotherapy and to base decisions upon that assessment.

Methods of assessment of response to therapy other than clinical examination include mammography and ultrasound. Baseline mammography provides initial information about the presence and extent of malignant calcification and also the presence of multifocal or contralateral carcinoma. However, as a method for assessing response to PMT it is less useful, as continuing mammographic density and calcification are common and correlate poorly with pathological findings (Moskovic et al, 1993).

Ultrasound is fast, safe, widely available and inexpensive. With the introduction of high-frequency, high-resolution transducers it has become an effective and increasingly used method for the primary assessment and follow-up of breast cancer (Balu-Maestro et al, 1991; Forouhi et al 1994; Tohno et al, 1994; Gawne-Cain et al, 1995). In addition, various workers have recently described the use of colour Doppler ultrasonography as an adjunct in the diagnosis of breast cancer by detecting malignant neovascularity both in the primary tumour and in axillary lymphadenopathy (Castagnone et al, 1993; Grishke et al, 1994; Kedar et al, 1994; Walsh et al, 1994). All these features make serial ultrasound scanning a useful method of monitoring response to chemotherapy, but the interpretation of residual sonographic abnormalities after treatment remains uncertain.

Accordingly, this study was undertaken to determine the role of ultrasound in monitoring response to primary, in particular, to

*Received 4 March 1996*
*Revised 19th February 1997*
*Accepted 26th February 1997*

*Correspondence to:* IE Smith

**Table 1** Patient characteristics ($n = 52$)

| | |
|---|---|
| Age | 26–60 years (median 45) |
| Menses | |
| Premenopausal | 34 |
| Peri/post-menopausal | 18 |
| Surgical assessment at presentation | |
| Operable by mastectomy | 52 |
| Suitable for conservation | 0 |
| Clinical TNM staging | |
| T2 (but > 4 cm) | 19 |
| T3 | 29 |
| T4 (operable) | 4 |
| N0 | 30 |
| N1 | 22 |
| M0 | 52 (minimal staging) |
| Histology (pretreatment core-cut biopsy) | |
| Grade 1 | 1 |
| Grade 2 | 17 |
| Grade 3 | 22 |
| Insufficient for grading | 12 |

**Table 2** Clinical and sonographic response to PMT

| | Clinical response | | | Total |
|---|---|---|---|---|
| | CR | PR | NC | |
| US response | | | | |
| CR | 5 | – | – | 5 (10%) |
| PR | 25 | 15 | – | 40 (77%) |
| NC | 1 | 5 | 1 | 7 (13%) |
| Total | 31 (60%) | 20 (38%) | 1 (2%) | 52 (100%) |

**Table 3** Clinical and pathological response to PMT

| | Clinical response | | | Total |
|---|---|---|---|---|
| | CR | PR | NC | |
| Pathological response | | | | |
| Not done | 7 | – | – | 7 (13%) |
| pCR | 9[a] | – | – | 9 (17%) |
| Non-invasive/ microinvasive | 8 | 6 | – | 14 (27%) |
| Macroinvasive carcinoma | 7 | 14 | 1 | 22 (42%) |
| Total | 31 (60%) | 20 (38%) | 1 (2%) | 52 (100%) |

[a]Of the nine pCRs, four were diagnosed from multiple core-cut biopsies, five from wide local excision.

improve our understanding of the nature of residual sonographic abnormalities at the end of chemotherapy.

### PATIENTS AND METHODS

Over the past 3 years, patients under 65 years old presenting to the Royal Marsden Hospital with large (≥ 4 cm), operable primary breast cancer have participated in a programme of primary medical therapy (PMT). A total of 52 such patients have been serially assessed by manual palpation and by colour Doppler ultrasound, and form the basis of this report. Their characteristics, and the characteristics of their tumours, are given in Table 1.

### Treatment and assessment protocol

In all cases, the diagnosis of invasive carcinoma was confirmed by core-cut needle biopsy before initiating treatment. PMT consisted of epirubicin, cisplatin and protracted infusional 5-fluorouracil (ECisF) as previously described (Jones et al, 1994; Smith et al, 1995), for 6–8 cycles of 21 days. In brief, this regimen comprises epirubicin 50–60 mg $m^{-2}$ and cisplatin 60 mg $m^{-2}$ on day 1, with continuous ambulatory venous infusion of 5-fluorouracil 200 mg $m^{-2}$ per 24 h throughout the cycle (days 1–21).

Bidimensional estimation of tumour size was made by a senior clinician using callipers before each cycle of chemotherapy (i.e. 3-weekly) and at the end of treatment. Ultrasonography was performed before treatment and 6-weekly thereafter. Although these assessments were not formally 'blinded', clinical assessments were routinely performed without knowledge of that day's ultrasound result and vice versa. Mammography was performed before treatment but, after work within the unit that showed little value of serial mammography in this circumstance (Moskovic et al, 1992), serial and post-treatment mammography were not routinely performed.

After primary medical therapy, patients were treated according to the residual abnormality. Those with no palpable abnormality were eligible to be treated with radiotherapy alone, although in most cases if a residual area of abnormality was detectable on ultrasound this was first widely excised under US-guided needle localization, or biopsied with multiple core-cut biopsies. Those with a residual palpable abnormality were subjected to either breast-conserving surgery or mastectomy according to the normal surgical criteria of size and position. Patients undergoing breast surgery also had level 2 axillary dissection.

All patients who had not undergone mastectomy received radiotherapy to the residual breast tissue. Those who had not had axillary dissection also received radiotherapy to the axilla. Other nodal areas were not routinely irradiated. All patients regardless of age, menstrual status and hormone receptor status received adjuvant tamoxifen 20 mg o.d. for 2 years from completion of chemotherapy.

### Ultrasound technique

Patients were scanned using a 7-MHz hand-held linear array transducer attached to an Acuson 128 computed sonography unit (Mountain View, CA, USA). Measurements of the tumour size (cm) in three orthogonal planes were obtained using real-time ultrasound and then the mass was assessed for colour Doppler signals. The Acuson capability for colour Doppler (5 MHz) was used for flow evaluation using the standardized machine settings. These settings were chosen to optimize sensitivity to low velocity and low-volume blood flow. Doppler positivity was ascribed if colour Doppler signals were obtained within 5 mm of the margin of the tumour or inside it. In addition, a visual score from 0 to 3 of the vascularity of the tumour provided by the colour Doppler signal was made at each scan.

After therapy, in the case of sonographic resolution of the primary breast tumour, the presence, appearance and measurements of any residual parenchymal distortion are recorded with any associated Doppler signal, if present.

© Cancer Research Campaign 1997

**Figure 1** **(A)** and **(B)** Pre- and post-treatment ultrasound appearances. This patient's tumour underwent clinical complete response. The residual sonographic abnormality **(B)** is a small area of parenchymal distortion (arrowed) from multiple core-cut biopsies were obtained under ultrasound guidance. **(C)** Histology showing foci of degenerate atypical cells highly suggestive of residual carcinoma

### Response criteria

For clinical and sonographic criteria, the UICC designations of treatment response were used, i.e. CR (complete response), no detectable tumour; PR (partial response), ≥ 50% reduction in bidimensional product; NC (no change), reduction of < 50% or increase of < 25% in bidimensional product; PD (progressive disease), increase of ≥ 25% in tumour bidimensional product. A clinical finding of 'vague thickening' in the vicinity of the breast previously occupied by tumour was scored as PR, as was the sonographic finding of 'vague parenchymal distortion'.

### RESULTS

The clinical and sonographic response rates are shown in Table 2. The ECisF chemotherapy regimen produced a high clinical CR (cCR) rate of 60%, but ultrasonographic abnormalities remained in the majority of these patients. A consistent finding in the general appearance of the post-treatment breast on ultrasound was the presence of diffusely echogenic tissue at the site of previous tumour and surrounding any residual tumour, which effaced the normal parenchymal echopattern locally. This brightly reflecting tissue appeared to correspond to hyalinized fibrofatty tissue seen pathologically in association with the region of treated tumour.

Post-treatment histological findings in 45 patients are shown in Table 3. Pathological CR (pCR) was found in nine, with a further 14 patients showing abnormalities not amounting to residual invasive tumour masses (scattered atypical cells in five; DCIS in four; microinvasion in five). The remaining 22 patients had residual invasive tumour masses.

### The contribution of ultrasound in patients without cCR

Twenty-one patients had palpable abnormalities at the end of treatment. In every case, surgery was performed (wide local excision in 18; mastectomy in three). DCIS alone was found in three, and microinvasion in two. The remaining 16 operative specimens contained residual macroscopic invasive cancers, although in five cases the residual disease was of a lower grade than the pretreatment biopsy.

In all 21 patients with palpable residual disease, a measurable sonographic lesion was also present. However, the ultrasound findings, including Doppler signal, were not helpful in distinguishing those patients with macroscopic invasive cancers from those with lesser histological findings (Table 4). Nor was ultrasound superior to clinical assessment in predicting the maximum diameter of the excised tumour (data not shown).

### The contribution of ultrasound in patients with cCR

Of the 31 patients with cCR, 15 underwent US-assisted wide local excision of sonographic abnormalities and nine had multiple US-guided core-cut biopsies (seven went on to radiotherapy without biopsy). Histology in these 24 patients showed residual macroscopic invasive cancer in six, microinvasion or scattered suspicious cells in six, DCIS in two, and no evidence of disease in ten. Table 5 shows the ultrasound and histological findings in these women.

The significance of normalization of the ultrasound (uCR) was not determined in this study. This occurred in five patients, but in only one was biopsy undertaken – using multiple core-cut biopsies of the previously affected segment. These showed no evidence of disease. Other patients with uCR proceeded to radiotherapy without biopsy.

In five patients the post-treatment ultrasound showed a residual area of diffuse parenchymal abnormality without Doppler signal. Histological findings in these patients varied from pCR in one patient to scattered degenerate carcinoma cells in three patients, to frank residual carcinoma in one patient. Figure 1 shows representative appearances in a patient with cCR whose post-treatment ultrasound showed diffuse parenchymal distortion. When multiple core-cut biopsies were obtained from this area, they showed microscopic foci of scattered degenerate atypical cells indicating residual carcinoma.

The remaining 21 patients with cCR had measurable residual mass lesions on ultrasound (Table 5). The presence of Doppler signal appeared to predict for residual disease in these patients: five out of six patients with Doppler positivity had residual cancer or DCIS on histology. On the other hand, among patients with Doppler-negative mass lesions, 7 out of 12 had pCR.

### DISCUSSION

The initial results of primary medical therapy (PMT) for large operable breast cancer are encouraging: the technique appears to enable breast-conserving treatment to be offered to a majority of the women who currently require mastectomy. The early results of our series and others suggest that this is not at the cost of a high rate of local relapse. It is not yet known whether PMT also confers an advantage in terms of long-term systemic control and survival; this question has been addressed in ongoing randomized trials by the NSABP, the EORTC and others. However, even if these trials were to show no overall survival advantage for PMT, the benefits

**Table 4** Ultrasound and pathological finding in patients without clinical CR

| Patients without clinical CR ($n = 21$) | US findings | | |
|---|---|---|---|
| | PR mass Doppler – | PR mass Doppler + | NC mass Doppler + |
| Pathological response | | | |
| Non-invasive/microinvasive | 3 | 3 | – |
| Macroinvasive carcinoma | 6 | 3 | 6 |

**Table 5** Ultrasound and pathological findings in patients with clinical CR

| Patients with clinical CR ($n = 31$) | US findings | | | |
|---|---|---|---|---|
| | CR | Parenchymal distortion | PR mass Doppler – | PR/NC mass Doppler + |
| Pathological response | | | | |
| Not done | 4 | – | 2 | 1 |
| pCR | 1 | 1[a] | 7[b] | 1 |
| Non-invasive/microinvasive | – | 3 | 2 | 3 |
| Macroinvasive carcinoma | – | 1 | 3 | 2 |

[a]Diagnosed by multiple core-cut biopsies. [b]Of the seven pCRs, four were diagnosed from multiple core-cut biopsies, three from wide local excision.

© Cancer Research Campaign 1997

of breast conservation will probably ensure continued interest in the approach.

In the authors' experience, PMT has been made more practicable by the development of the highly effective ECisF chemotherapy regimen. This has permitted the treatment of patients with large tumours, at the borderline of operability, with a negligible risk of progressive disease during treatment and a high probability of clinical complete response (cCR). A multicentre randomized trial is under way in the UK comparing this new regimen with a conventional chemotherapy schedule (doxorubicin, cyclophosphamide; AC) as PMT for operable breast cancer.

The use of chemotherapy to shrink primary tumours leads to new, welcome but difficult decisions in subsequent surgical management. The key questions are: what is the minimum and most conservative surgery that may be performed (with post-operative radiotherapy), without risk of local relapse, after a large tumour has been 'downsized' by PMT; and under what circumstances might surgery be omitted altogether?

The current working assumption of our unit and others is that, after primary chemotherapy, residual invasive carcinoma, microinvasive carcinoma or carcinoma in situ should be widely resected and the breast subsequently irradiated. Conversely, patients with pathological CR might safely be treated with radiotherapy alone. However, it is not known whether these assumptions are correct. Treatment of primary DCIS with radiotherapy is an accepted alternative to mastectomy, forming an arm of current trials, and a similar approach might reasonably be considered with non-invasive carcinoma, or even microinvasive carcinoma, after primary chemotherapy.

The use of histological criteria for guiding post-PMT management raises its own problems. In this study, a compromise was reached between the certainty of the histological diagnosis and the demands of breast conservation. For those patients in whom PMT was most effective and the tumour site was no longer easily identifiable after treatment, an extensive resection would have been required for full histological assessment. Instead, we used the approach of more limited sampling with multiple core-cut biopsies, or in some cases no biopsy. Inevitably, this limits the confidence of our histological response rate. Some other centres use skin tattooing or inject carbon or metal markers into the tumour at the start of treatment in order to allow selective biopsy of the area after PMT (Veronesi et al, 1995).

The potential role of ultrasound in post-chemotherapy assessment is to help select, in cases where there is clinical doubt, those patients who do or do not require excision, and to localize lesions in those who do.

For patients who had a residual palpable tumour or 'thickening' at the end of PMT, we found that US contributed little. All these patients had residual pathological lesions that by our current criteria required excision, although in six cases these did not amount to macroscopic invasive carcinoma. In all cases, sonographic lesions persisted, but the sonographic findings (including Doppler positivity) were not required for localization, did not contribute to the surgical decision and did not identify the patients with only non-invasive or microinvasive residual disease.

In patients with clinical CR (cCR), ultrasound proved a more useful investigation. The majority (84%) of patients with cCR had residual sonographic abnormalities; ultrasound is a useful technique for localization in these patients, but does it predict the pathological findings and identify a group who may not require surgery? Of the 23 such patients who underwent guided excision or biopsy, nine (39%) proved to have pathological CR (pCR) and in all but one of these patients the sonographic findings had been of small, Doppler-negative lesions or parenchymal distortion rather than a Doppler-positive mass. However, these 'favourable' sonographic features were also seen in nine other patients who did not have pCR. The small group of patients with complete resolution of the sonographic abnormality (uCR) probably represent those with the most favourable response to PMT. Histological assessment of these patients was not undertaken in this study, for the reasons already discussed, but it seems reasonable to adopt the approach that patients with sonographic CR have the highest probability of pCR or minimal residual disease and are adequately managed with radiotherapy alone.

In this study, the strength of colour Doppler positivity (i.e. vascularity) was assessed visually but not quantitatively. This may have limited our ability to correlate Doppler findings with ultimate histology. Other workers, who have measured Doppler positivity quantitatively using colour-capture techniques, have recently reported excellent correlation between the reduction in sonographic tumour size and the reduction in Doppler signal during primary medical therapy (Kedar et al, 1994). This and other new refinements in ultrasound technology may be useful for the detection of abnormal low-velocity flow in vessels. A recent report has shown dedicated breast magnetic resonance imaging (RODEO MRI) to be a promising technique for prediction of pathological response to primary chemotherapy (Abraham et al, 1996), and a comparison of these techniques would be interesting.

The next step in rationalizing decisions about treatment after PMT must involve randomization between different approaches. For example, patients with cCR after PMT could be randomized to have radiotherapy alone or US-guided wide local excision plus radiotherapy. The reference of ultrasound assessment will be determined by long-term follow-up from such randomized trials.

### ACKNOWLEDGEMENT

The authors wish to thank Fiona Bolton for her help in preparing the manuscript.

### REFERENCES

Abraham DC, Jones RC, Jones SE, Cheek JH, Peters GN, Knox SM, Grant MD, Hampe DW, Savino DA and Harms SE (1996) Evaluation of neoadjuvant chemotherapeutic response of locally advanced breast cancer by magnetic resonance imaging. *Cancer* **78**: 91–100

Balu-Maestro C, Bruneton J-N, Goeffray A, Chauvel C, Rogopoulos A and Bittman O (1991) Ultrasonographic post-treatment follow-up of breast cancer patients. *J Ultrasound Med* **10**: 1–7

Bonadonna G, Veronesi U, Brambilla C, Ferrari L, Luini A, Greco M, Bartoli C, Deyoldi GC, Zuchali R, Rilke F, Andreola S, Silvestrini R, Difronzo G and Valgussa P (1990) Primary chemotherapy to avoid mastectomy in tumours with diameters of three centimetres or more. *J Natl Cancer Inst* **82**: 1539

Castagnone, D, Rescalli, S, Rivolta, R, Burdick, L, Nosotti, M, and Poma, S (1993) Colour Doppler ultrasound in the diagnosis of a solid breast mass. *Breast* **2**: 115–117

Fisher B, Rockette H, Robidoux A, Margolese R, Cruz A, Hoehn J, Boysen D, Mamounas E, Wickerham DL and Decillis A (1994) Effect of preoperative therapy for breast cancer on local-regional disease: first report of NSABP B-18 (abstract no. 57). *Proc Am Soc Clin Oncol* **13**: 64

Forouhi P, Walsh JS, Anderson TJ and Chetty U (1994) Ultrasonography as a method of measuring breast tumour size and monitoring response to primary systemic treatment. *Br J Surg* **81**: 223–225

Gawne-Cain ML, Smith E, Darby M and Given-Wilson, R (1995) The use of ultrasound for monitoring breast tumour response to pro-adjuvant therapy. *Clin Radiol* **50**: 681–686

*© Cancer Research Campaign 1997*

Grischke EM, Kaufmann M, Eberlein-Gonska M, Mattfeldt T, Sohn CH, Bastert G (1994) Angiogenesis as a diagnostic factor in primary breast cancer: microvessel quantitation by stereological methods and correlation with color Doppler sonography. *Onkologie* **17**: 35–42

Jones AL, Smith IE, O'Brien MER, Talbot D, Walsh G, Ramage F, Robertshaw H and Ashley S (1994) Phase II study of continuous infusion fluorouracil with epirubicin and cisplatin in patients with metastatic and locally advanced breast cancer: an active new regimen. *J Clin Oncol* **12**: 1259–1265

Kedar RP, Cosgrove DO, Smith IE, Mansi JL and Bamber JC (1994) Breast carcinoma: measurement of tumor response to primary medical therapy with color Doppler flow imaging. *Radiology* **190**: 825–830

Moskovic EC, Mansi JL, King DM, Murch CR and Smith IE (1993) Mammography in the assessment of response to primary medical treatment of large primary breast cancer. *Clin Radiol* **47**: 339–344

Powles TJ, Hickish TF, Makris A, Ashley SE, O'Brien ME, Tidy VA, Casey S, Nash AG, Sacks N, Cosgrove D (1995) Randomized trial of chemoendocrine therapy started before or after surgery for treatment of primary breast cancer. *J Clin Oncol* **13**: 547–552

Smith IE, Walsh G, Jones AL, Prendiville J, Johnston S, Gunterson B, Ramage F, Robertshaw H, Sacks N, Ebbs S, McKinna JA and Baum M (1995) High complete remission rates with primary neoadjuvant infusional chemotherapy for large early breast cancer. *J Clin Oncol* **13**: 424–429

Tohno E, Cosgrove DO and Sloane JP (1994) Malignant disease – primary carcinomas. In *Ultrasound Diagnosis of Breast Diseases*. pp. 158–179, Churchill Livingstone, London.

Veronesi U, Bonadonna G, Zurrida S, Galimberti V, Greco M, Brambilla C, Luini A, Andreola S, Rilke F, Raselli R (1995) Conservation surgery after primary chemotherapy in large carcinomas of the breast. *Annals Surg* **222**: 612–618

Walsh JS, Dixon JM, Chetty U, Paterson D (1994) Colour Doppler studies of axillary node metastases in breast carcinoma. *Clin Radiol* **49**: 189–191

Weil M, Borel C, AuClerc G, Petit T, Rixe O, Thomas A, Kolodziejska E, Nizri D, Baillet F, Soubrane C and Khayat D (1995). Mature results in 477 breast cancer patients treated by neoadjuvant chemotherapy and exclusive radiotherapy (abstract 0-763). *Int Congr Anti-Cancer Chemother* **5**: 165

© Cancer Research Campaign 1997